# Painful stimulation increases spontaneous blink rate in healthy subjects

**DOI:** 10.1038/s41598-020-76804-w

**Published:** 2020-11-17

**Authors:** Giulia Paparella, Giulia Di Stefano, Alessandra Fasolino, Giuseppe Di Pietro, Donato Colella, Andrea Truini, Giorgio Cruccu, Alfredo Berardelli, Matteo Bologna

**Affiliations:** 1 IRCCS Neuromed, Pozzilli, IS Italy; 2 Department of Human Neurosciences, Sapienza University of Rome, Viale dell’Università, 30, 00185 Rome, Italy

**Keywords:** Neural circuits, Neuronal physiology, Somatosensory system

## Abstract

Spontaneous blink rate is considered a biomarker of central dopaminergic activity. Recent evidence suggests that the central dopaminergic system plays a role in nociception. In the present study, we aimed to investigate whether pain modulates spontaneous blink rate in healthy subjects. We enrolled 15 participants. Spontaneous blink rate was quantified with an optoelectronic system before and after: (1) a painful laser stimulation, and (2) an acoustic startling stimulation. In control experiments, we investigated whether laser stimulation effects depended on stimulation intensity and whether laser stimulation induced any changes in the blink reflex recovery cycle. Finally, we investigated any relationship between spontaneous blink rate modification and pain modulation effect during the cold pressor test. Laser, but not acoustic, stimulation increased spontaneous blink rate. This effect was independent of stimulation intensity and negatively correlated with pain perception. No changes in trigeminal-facial reflex circuit excitability were elicited by laser stimulation. The cold pressor test also induced an increased spontaneous blink rate. Our study provides evidence on the role of dopamine in nociception and suggests that dopaminergic activity may be involved in pain modulation. These findings lay the groundwork for further investigations in patients with pathological conditions characterized by dopaminergic deficit and pain.

## Introduction

Neurophysiological studies on blinking are a valid approach for the investigation of various anatomical and functional substrates in healthy subjects and in pathological conditions^1–4^. Spontaneous blink rate (SBR) is strictly related to dopamine levels in the central nervous system and is considered a reliable noninvasive biomarker of central dopaminergic activity^3–14^. Reduced SBR is a common finding in Parkinson’s disease (PD) and other parkinsonian syndromes characterized by dopaminergic loss^3,6–13^. Conversely, increased SBR has been observed in conditions characterized by increased dopaminergic tone, such as schizophrenia^14^. Increasing evidence indicates a possible relationship between the central dopaminergic system and pain modulation in both animal and human studies^15–19^. A subpopulation of dopaminergic neurons within the ventrolateral periaqueductal grey (PAG), a brain area included in the descending pain modulatory system^20^, projects to brain regions known to be involved in pain modulation^21–24^. Both D1-like and D2-like dopamine receptors are expressed within the ventrolateral PAG^25–27^ and have been shown to contribute to antinociception^15,28–30^.

In the present study, we aimed to investigate whether SBR, as an indirect marker of central dopaminergic activity, is modulated by pain in healthy subjects. For this purpose, we tested the effects of phasic pain produced by laser stimulation^31,32^ on SBR objectively quantified with a kinematic analysis system, as compared to effects induced by auditory startling stimulation^33^. We also investigated whether pain-induced SBR changes were due to modifications of SBR generator or trigeminal-facial reflex circuit excitability. For this purpose, we tested whether laser simulation changed the blink rate recovery cycle (BRRC)^34^. Lastly, we analyzed SBR during the cold pressor test, a widely agreed upon method to activate the descending pain modulatory system^32,35^. Clarifying these issues could provide important physiological information on the role of the dopaminergic system in nociception. Our results may also lead to a better understanding of the relationship between dopamine and pain in pathological conditions.

## Material and methods

### Participants

Fifteen healthy subjects were enrolled (8 females, mean age ± 1 SD: 26.6 ± 3.71 years; age range 23–37 years). This study was approved by the Institutional Review Board of ‘Sapienza, University of Rome’, and performed in accordance with the Declaration of Helsinki for the use of humans in experimental studies. Written informed consent was obtained from all participants. All subjects were right-handed (Table 1). The following exclusion criteria were considered: experiencing a preexisting pain condition, self-reported medication consumption, and the presence of any medical conditions, including neurological or psychiatric diseases.

**Table 1:** Demographic and clinical data.

	Gender	Age	Heat threshold	Nociceptive threshold	Laser stimulation intensity
1	M	25	51	76	178
2	M	32	51	76	178
3	M	27	51	76	178
4	M	25	51	101	203
5	F	25	51	76	178
6	F	29	25	51	152
7	F	24	25	76	178
8	F	29	25	76	178
9	F	24	51	76	203
10	M	37	76	101	203
11	M	25	51	76	178
12	F	25	51	76	152
13	F	25	25	76	203
14	F	23	51	101	178
15	M	25	51	76	178
Average		26.66	45.73	79.33	181.42
1 SD		3.71	14.42	12.90	16.84

F: female; M: male; SD: standard deviation. Age is expressed in years. The heat threshold, the nociceptive threshold and laser stimulation intensity are expressed in mJ/mm^2^.

### Kinematic recordings and blinking analysis

Participants were comfortably seated on a chair during kinematic recordings, with both arms slightly abducted from the trunk, elbows slightly flexed, and the dominant forearm resting on a table in a prone position. To record spontaneous blinking, participants were requested to relax and look straight forward. Blinking movements were recorded using an optoelectronic motion system (SMART Motion System, BTS Engineering, Milan, Italy), version number 1.10.0462, https://www.btsbioengineering.com/it/prodotti/smart-dx-2/. This system included three infrared cameras (120 Hz sampling rate) which followed the 3D space displacement of reflective markers of negligible weight taped on participants’ head^10–13^. Two markers were taped on the upper eyelids and three additional markers were placed over the frontal orbital processes (bilaterally) and one over the nasion, in order to derive the head coordinate system. An examiner visually inspected the traces before the automatic analysis, to exclude uncompleted recordings (less than 1% of the overall traces). The kinematic analysis of the SBR was then automatically performed by a dedicated software (SMART Analyzer, BTS, Milan, Italy), which defined the beginning and end of the closing and opening blink phases of a blink movement when the velocity first reached or returned to 10% of the peak velocity of each phase^10^. This methodological approach allowed us to reduce any possible bias due to a visual analysis of the blinking recordings^11^. The automatic system then calculated the SBR, i.e. the number of spontaneous blinks per minute for each recording block^10–13^. We also analyzed SBR in the time interval before (pre-SBR) and after (post-SBR) the stimulation for each block. Finally, we considered the interval between two spontaneous blinking movements (for each interval I1, I2, I3 … In). We then calculated the reciprocal of the interval (1/In) between two consecutive blinking movements to obtain an instantaneous SBR (I-SBR). We considered the I-SBR of the five blinking movements before and after laser and acoustic stimulation. Pre-SBR, post-SBR, and I-SBR represent stimulation effects on each recording block and were considered secondary outcomes.

To evoke blinking movements, we transcutaneously applied electrical stimuli to the right supraorbital nerve at an intensity of ~ 4 to 6 times the sensory threshold^4,9,10,36^. The BRRC protocol consisted of paired stimulations in which a conditioning stimulus was followed by a test stimulus with interstimulus intervals (ISIs) of 250 ms and 500 ms^34^. We recorded five blocks for ISI. The two ISIs were presented in randomized order and an intertrial interval of about 40–60 s separated the trials^4,10,10^ . The dedicated software (SMART Analyzer, BTS, Milan, Italy, version number 1.10.0462) was used to measure the peak velocity of the closing phases of unconditioned and conditioned responses^9–11^. We then calculated the ratio between conditioned and unconditioned responses as a measure of reflex response recovery. We selected the peak velocity of the closing phases because previous observations showed that it positively correlated with the size of orbicularis oculi muscle activation in response to supraorbital electric nerve stimulation^10,37^.

### Laser and acoustic stimulation

To induce pain, we stimulated the skin on the dorsum of the right forearm with a Neodymium‐YAP stimulator. Laser pulses were set with an intensity of 75–200 mJ/mm^2^, a duration of 5 ms, and a diameter of 5 mm^31^ in order to elicit a clear pinprick sensation in all subjects^38^ resulting in a subjective rating of at least 4 on a 0–10 numeric rating scale (NRS) (0 = no sensation, 10 = worst possible pain)^39,40^. As a perceptive threshold, we considered the value of the lowest stimulus intensity at which the subject perceived at least 50% of the stimuli as painful^41,42^.

The acoustic startling stimulation was applied binaurally (Sony Auricular MDR-201) with tone bursts of 120 dB SPL (ISO, frequency 1000 Hz, duration 120 ms)^33,43^.

### Cold pressor test

The water bath for the cold pressor test was a plastic box filled with ice and water^32,35^. We monitored water temperature during the entire duration of the experimental procedure with an electronic probe thermometer TFA Dostmann (4–7 °C)^44^. Subjects were asked to keep their right hand in the ice-cold water until they subjectively perceived pain that scored at least 6 on the NRS. SBR was recorded in blocks of 180-s duration.

The pressure pain threshold (PPT) was also measured on the right arm during the cold pressor test with a pressure gauge device (FDN200, Wagner Instruments, USA) with a probe area of 1 cm^2^ (probe diameter of 1.1 cm) that exerted forces up to 20 kg/cm^2^, corresponding to 2000 kPa. PPT was considered a measure of descending pain modulatory system activation.

### Experimental design

The two sessions of the main experiment were randomly performed at least one week apart. In each session, we recorded 10 blocks of 180 s each. A single laser or acoustic stimulus was delivered at 90 ± 3 s in each recording block. A rest interval of 30 s was administered between blocks. After each stimulus, subjects were asked to quantify the intensity of perception from 0 to 10 on the NRS scale^39,40^.

In the first control experiment, performed on 10 of the 15 subjects, we tested whether SBR modulation induced by laser stimulation depended on stimulation intensity. We compared the effects of two laser stimulation intensities: low (slightly above the pain threshold) and high intensity (two-and-a-half times the pain threshold). Five randomly ordered SBR recordings of 180 s were performed for each intensity.

In the second control experiment, performed on 9 of the 15 subjects, we tested whether SBR modulation was due to concomitant brainstem excitability changes. For this purpose, we applied the BRRC protocol before (pre) and immediately after (post) five laser stimulation blocks of 180 s, as in the main experiment. We acquired five laser stimulation blocks, which were sufficient to detect significant SBR changes. In each measurement time lapse (pre and post), we performed 10 kinematic recordings of the BRRC (five conditioned with an ISI of 250 ms, five conditioned with an ISI of 500 ms), delivered in a random order.

In the third control experiment, performed on 9 of the 15 subjects, we tested SBR (five recording blocks of 180 s) before (T0), during (T1), and 5 (T2), 15 (T3), and 45 min (T4) after the cold pressor test. The test consisted of the immersion of the right hand in ice water. This tonic painful stimulus is known to activate the descending pain modulatory system^17,45^. This control experiment was designed to test the effect of this tonic model on SBR and to explore the possible implications of the descending modulatory system on SBR changes. To demonstrate effective activation of the descending control system, we measured PPT by applying the pressure gauge device on the right arm at each time point.

### Statistical analysis

The effects of laser and acoustic stimulation on SBR and/or the NRS were evaluated using non-parametric Friedman analysis of variance (ANOVA) for repeated measures with the classification factor RECORDING BLOCK (10 levels: blocks 1–10). The slopes of the regression lines reflecting SBR modulation were compared using Wilcoxon signed-rank test. We also compared pre-SBR and post-SBR of laser and acoustic startling sessions with Wilcoxon signed-rank test. We analyzed possible I-SBR changes by comparing the five intervals before and after laser and acoustic startling stimulation through Friedman ANOVA with the factor INTERVAL (10 levels).

To investigate possible differences in the effects of laser stimulation delivered at different intensities in the first control experiment, we compared SBR values during the low- and high-intensity recording blocks with Wilcoxon signed-rank test. We also compared pre-SBR and post-SBR for each stimulation intensity with Wilcoxon signed-rank test.

To evaluate BRRC variations before and after the five laser stimulation blocks, we included the ratios between the conditioned and unconditioned responses in repeated measures (rm) ANOVA with the factors TIME POINT (two levels: pre and post) and ISI (two levels: 250 ms and 500 ms). Finally, to evaluate SBR variations due to the cold pressor test, we used Friedman ANOVA with the classification factor TIME POINT (five levels: T0, T1, T2, T3, and T4). Post-hoc analyses on Friedman ANOVA were performed with Wilcoxon signed-rank test. Post-hoc analyses on rmANOVA were performed with the t-test. Results were corrected for multiple comparisons using the false discovery rate (FDR)^46^.

The Pearson’s product-moment correlation coefficient was calculated to evaluate possible associations between nociception thresholds, individual changes in SBR, and pain perception, as assessed by NRS and PPT values. For this purpose, we used linear regression techniques to determine the slope of the regression line that reflected the modulation of pain quantified with SBR as a function of the recording blocks. For PPT, we considered the ratio between PPT before and during the cold pressor test. Unless otherwise indicated, all results are shown as mean values ± 1 standard error of the mean (SEM). In all tests, the significance level was set at *P* < 0.05. Data were analyzed using STATISTICA (TIBCO Software Inc., Palo Alto, California, USA), version number 10, https://www.tibco.com/.

## Results

### Main experiment

**Figure 1:**
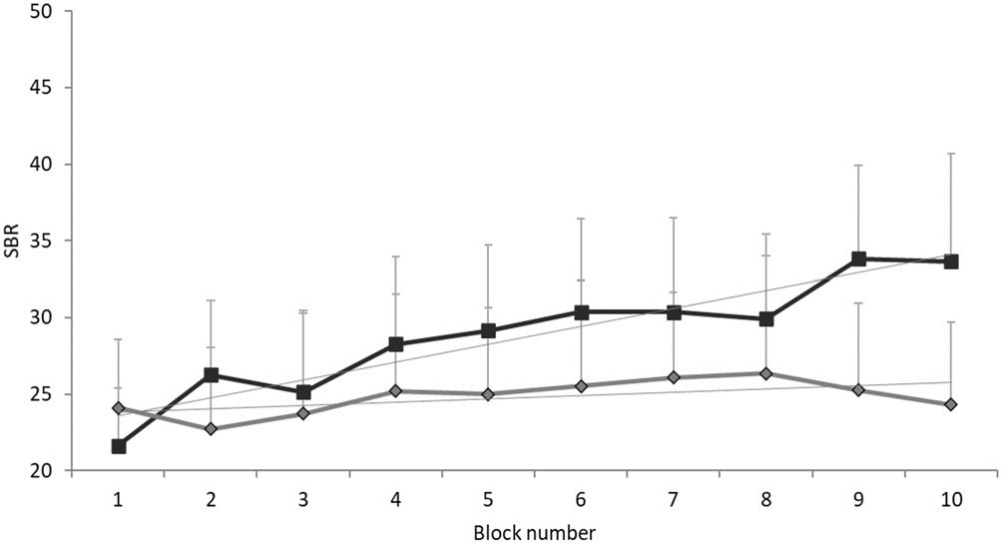
Overall spontaneous blink rate. Overall spontaneous blink rate (SBR) (Y axis) across the 10 recording blocks (X axis) of the two sessions of the main experiment. Blink rate is expressed as the average for each recording block. Black indicators represent the laser stimulation blocks while light grey indicators represent the acoustic blocks. Error bars denote standard errors.

**Figure 2:**
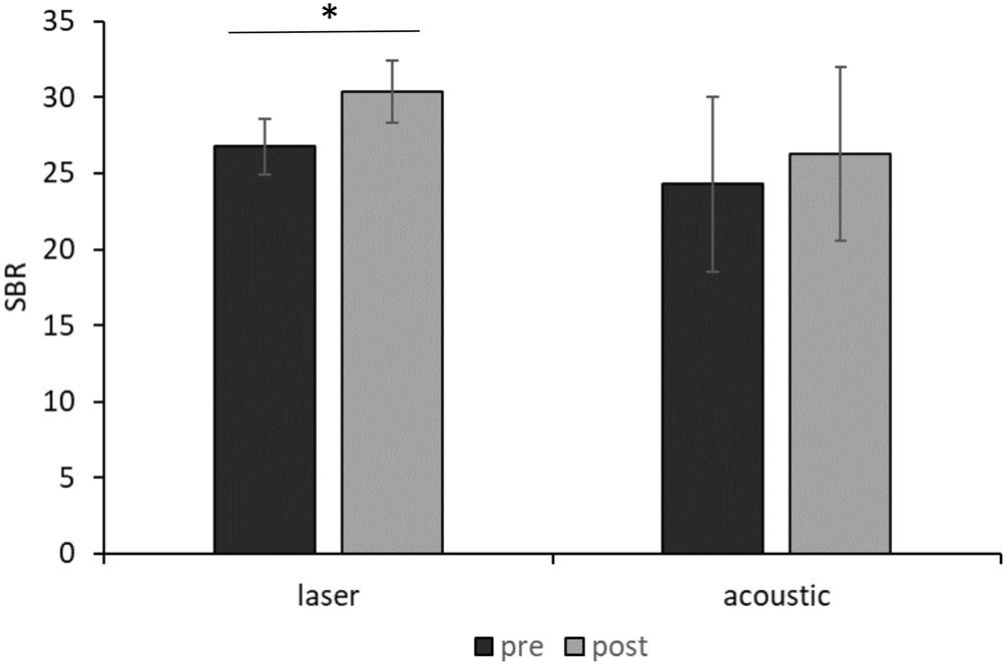
Spontaneous blink rate modifications. Spontaneous blink rate (SBR) recorded before (pre) and after (post) the laser or the acoustic stimulations in the two main experimental sessions. Error bars denote standard errors. Asterisks indicate *P* < 0.05 in the post-hoc comparisons.

**Figure 3:**
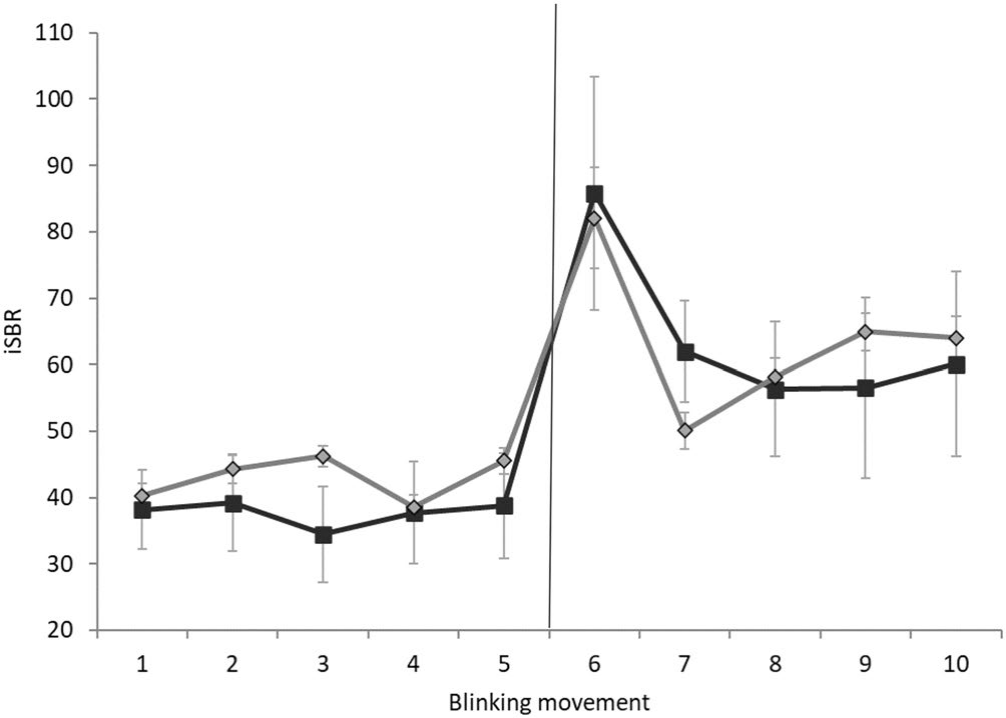
Instantaneous blinking frequency. Instantaneous blinking frequency (I-SBR) of the five blinking movements before and after the laser or the acoustic interventions in the main experiment was calculated as the reciprocal of the interval between two consecutive blinking movements. Black indicators represent the laser stimulation blocks while light grey indicators represent the acoustic blocks. Error bars denote standard errors. The vertical line indicates the time point at which the laser and acoustic stimulation were delivered. Note that with respect to the considered baseline values before stimulation (that is the 5th I-SBR) there was an increase of the I-SBR in both laser and acoustic stimulation sessions.

None of the participants reported adverse effects during or after the experimental procedures. SBR increased during laser, but not acoustic, stimulation (Fig. 1) as demonstrated by Friedman ANOVA, which showed a significant effect of the factor RECORDING BLOCK for the laser stimulation session (*X*_15,9_ = 28.01, *P* < 0.01). Post-hoc analysis revealed higher values in the 5th to 10th recording blocks in comparison with the 1st acquisition block (all *P* values ≤ 0.026, with 0.033 corrected α level by FDR). Conversely, no significant effect of the factor RECORDING BLOCK emerged from the ANOVA for acoustic stimulation (*X*_15,9_ = 8.38, *P* = 0.49). In addition, the slope of the SBR curves across the laser and acoustic recording blocks significantly differed (*P* = 0.03). Thus, SBR progressively increased across the 10 blocks of the laser, but not acoustic startling stimulation session. Furthermore, post-SBR values of the laser session were significantly higher than pre-SBR values (*P* = 0.01), while there was no difference between pre-SBR and post-SBR of the acoustic startling stimulation session (*P* = 0.21) (Fig. 2). Finally, Friedman ANOVA of I-SBR showed a significant difference between recording block values in both laser and acoustic startling sessions (*X*_15,19_ = 38.79, *P* < 0.01 for the laser session and *X*_15,19_ = 20.65, *P* = 0.01 for the acoustic startling session). Post-hoc comparisons revealed a temporary increase of the I-SBR immediately after the laser and acoustic startling stimulations. The I-SBR of the 6th and 7th blinking movements (i.e. the first two blinking movements after the laser stimulation) and the I-SBR of the 6th blinking movement (i.e. the first blinking movement after acoustic stimulation) differed from the I-SBR of the 5th blinking movement (i.e. the first blinking movement before laser or acoustic stimulation), which was considered the baseline value (all *P* values ≤ 0.007, with 0.011 corrected α level by FDR) (Fig. 3).

### Control experiments

**Figure 4:**
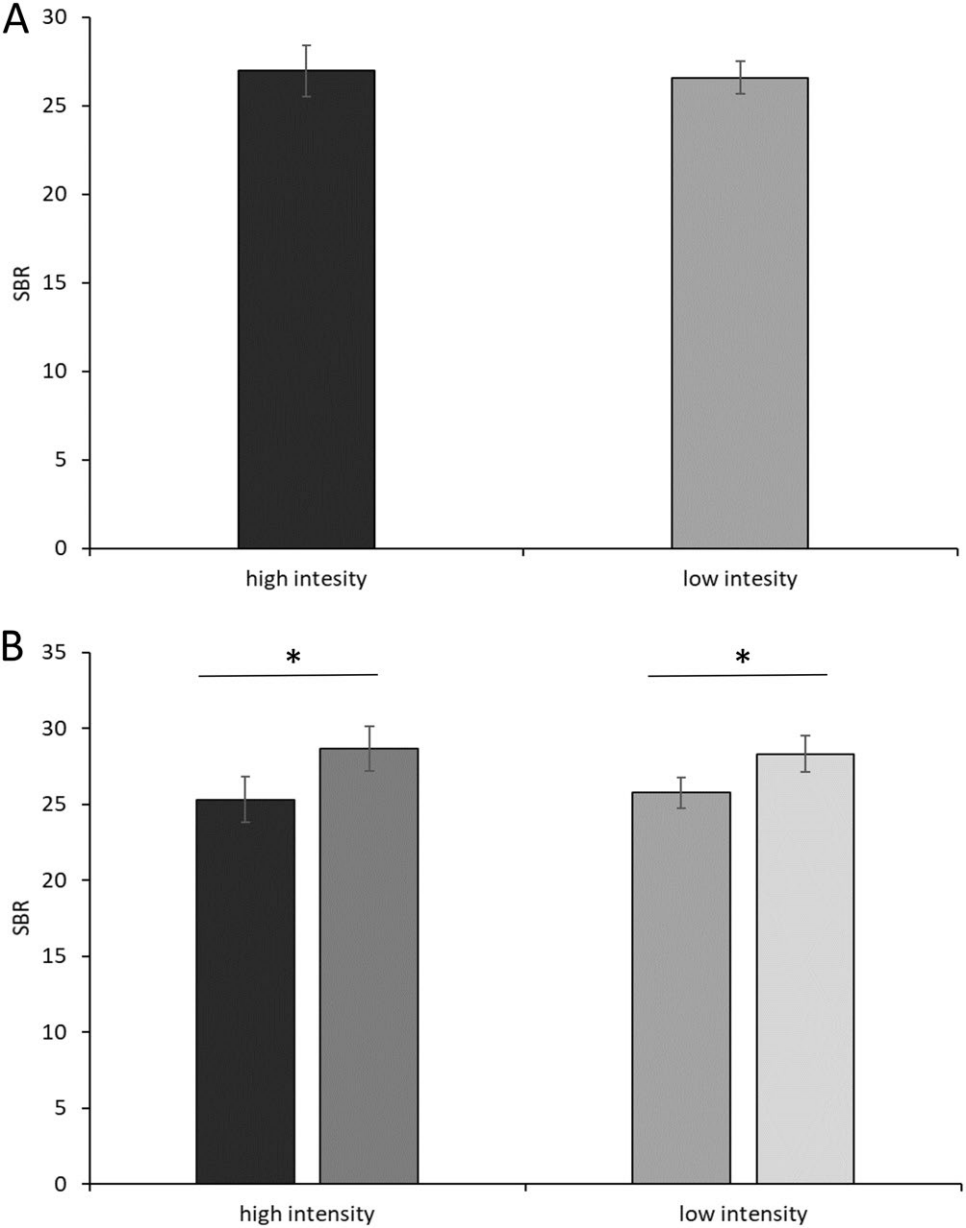
Effect of laser stimulation intensity on the spontaneous blink rate. (**A**) Overall spontaneous blink rate (SBR) recorded during the high-intensity and low-intensity stimulation blocks of the first control experiment. (**B**) SBR recorded before (dark grey) and after (light grey) the high- and low-intensity laser stimulation in the first control experiment. Error bars denote standard errors. Asterisks indicate *P* < 0.05 in the post-hoc comparisons.

SBR analysis during the low- and high-intensity recording blocks showed no differences between conditions (*P* = 0.5) (Fig. 4A). The comparison between pre-SBR and post-SBR showed a significant difference between values for both low- and high-intensity laser stimulation (*P*s = 0.043) (Fig. 4B).

**Figure 5:**
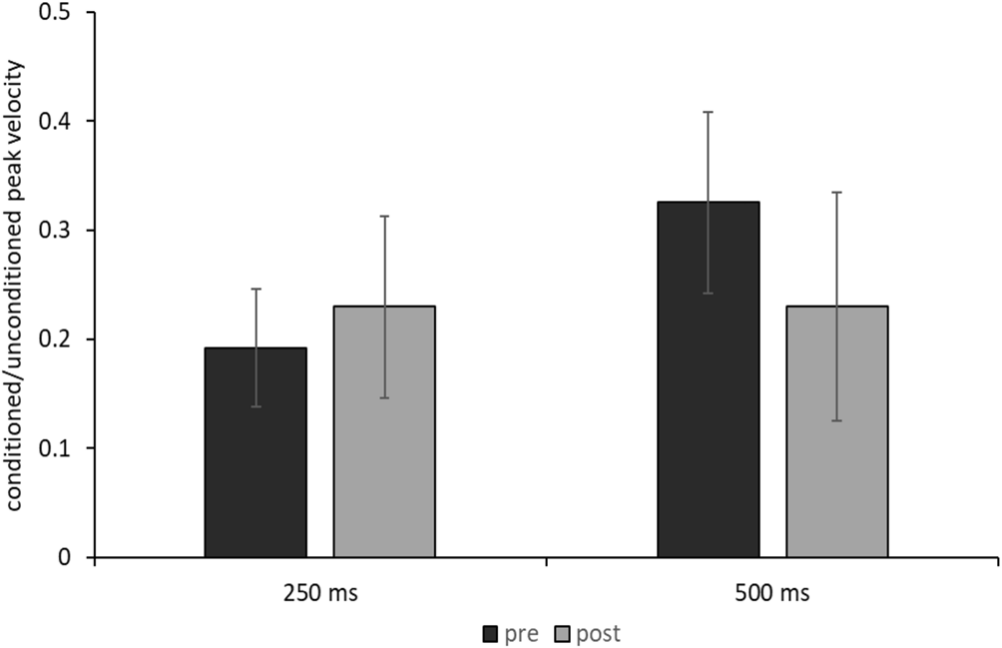
Blink reflex recovery cycle before changes due to the laser stimulation. Bars indicate the average of the ratios between the peak velocity of the closing phase of the conditioned responses and the peak velocity of the closing phase of the unconditioned responses before (pre) and after (post) 5 blocks of laser stimulation across 8 subjects, using an ISI of 250 ms (on the left) and 500 ms (on the right).

**Figure 6:**
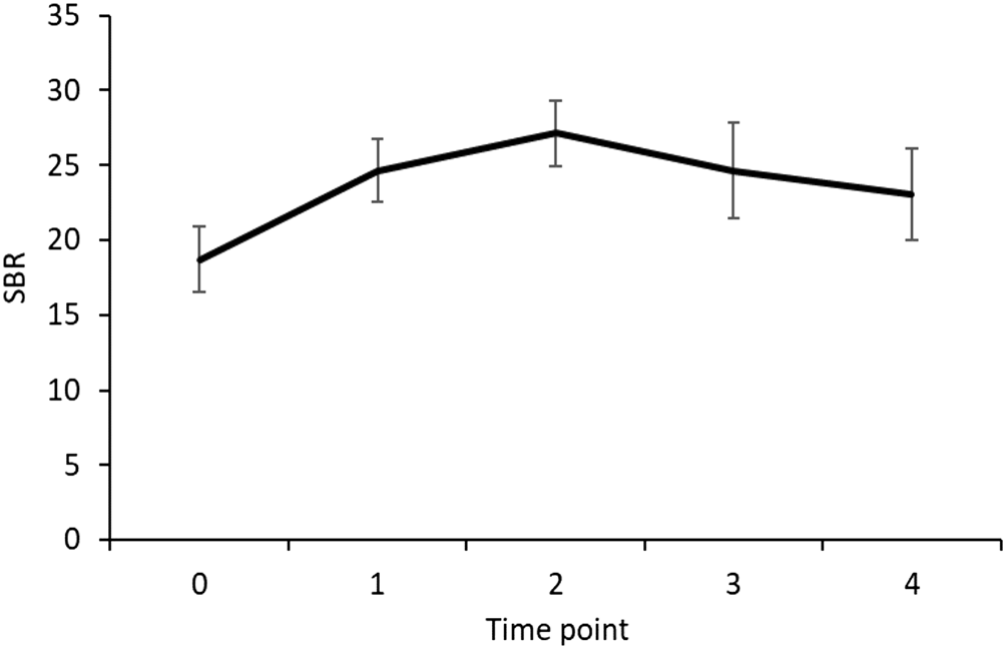
Spontaneous blink rate across the cold pressor test. Overall spontaneous blink rate (Y axis) across the cold pressor test session. Data indicate the blink rate average of the 10 subjects before (T0), during (T1), and 5 min (T2), 15 min (T3), and 45 min (T4) after the cold pressor test. Error bars denote standard errors.

Analysis of possible BRRC variations due to laser stimulation (Table 2) did not reveal any significant effects of the factor TIME POINT (F_1, 7_ = 0.42192, *P* = 0.84) or of the interaction TIME POINT x ISI (F_1, 7_ = 0.30503, *P* = 0.59). The analysis showed, as expected, a significant effect of the factor ISI (F_1, 7_ = 23.973, *P* < 0.01), indicating lower peak velocity values of conditioned responses for ISI 250 ms (Table 2). Notably, Friedman ANOVA on SBR in the control experiment showed a significant effect of the factor RECORDING BLOCK (*X*_9,4_ = 11.91, *P* = 0.018) and post-hoc analysis showed that the 5th SBR block differed from the 1st SBR acquisition block (*P* = 0.038). Thus, although laser stimulation induced an increase in SBR, it did not cause any changes in trigeminal-facial reflex circuit excitability (Fig. 5). Finally, SBR increased overall across the blocks recorded in the cold pressor test session (Fig. 6). The Friedman ANOVA showed a significant effect of the factor TIME POINT (*X*_9,4_ = 12.49, *P* = 0.014). Post-hoc analysis revealed that SBR values were significantly higher in T1 and T2 in comparison with T0 (all *P* values ≤ 0.025, with 0.037 corrected α level by FDR). Thus, the cold pressor test modified SBR, which progressively increased during the test, and the returned to baseline values. The PPT before the cold pressure test was 6.64 ± 1.53 kPa. The PPT during the cold pressor test was 8.44 ± 2.25 kPa. Thus, the PPT ratio was 1.29 ± 0.14.

**Table 2:** Blink reflex recovery cycle.

	ISI 250 ms			ISI 500 ms		
	Unconditioned	Conditioned	Ratio	Unconditioned	Conditioned	Ratio
PRE	257.14 ± 63.45	48.43 ± 15.26	0.19 ± 0.15	262.46 ± 76.69	129.29 ± 65.48	0.51 ± 0.23
POST	236.32 ± 35.29	52.98 ± 18.79	0.24 ± 0.23	254.19 ± 89.79	106.41 ± 95.51	0.49 ± 0.29

The peak velocities of the closing phase of the unconditioned and conditioned responses for the interstimulus intervals (ISIs) of 250 and 500 ms are expressed in mm/s before (PRE) and after (POST) the 5 laser stimulation blocks. Ratios are between the conditioned and unconditioned responses. Results are shown as mean values ± 1 standard deviation (SD).

### Correlation analysis

Pearson’s product-moment correlation showed no relationship between individual nociceptive thresholds and SBR changes. Notably, the analysis showed an inverse correlation between individual SBR changes induced by laser stimulation and pain perception changes, as assessed by the NRS (r = − 0.56; *P* = 0.03). Correlation analysis for the cold pressor test session revealed no correlations between SBR changes, NRS values, and PPT ratio.

## Discussion

In the present work, we used neurophysiological techniques to investigate the effects of painful stimulation on SBR. Our results demonstrated for the first time that both phasic and tonic painful stimulation induce a progressive and specific increase in SBR without modifying BRRC, indirectly reflecting an increased central dopaminergic tone^2,3,9,14^. The effects on SBR were unrelated to the intensity of painful stimulation, negatively correlated with changes of pain perception, and can be possibly explained by the activation of the descending pain modulatory system. The latter hypothesis is supported by the observation that SBR increased during the cold pressor test and lasted beyond the end of the tonic stimulus.

There are several physiological interpretations of SBR changes due to painful stimulation. One possibility is that SBR increase is due to nonspecific factors, e.g. modifications in arousal state induced by an unexpected stimulus. However, this hypothesis can be excluded in light of the results of the acoustic stimulation session. Although acoustic stimulation induced an increase in I-SBR similar to laser stimulation, thus confirming that both stimulations were startling, acoustic stimulation did not induce any SBR changes across blocks. Finally, the lack of concomitant pain-induced BRRC modification excluded that blink rate changes were due to brainstem excitability variations. In this regard, previous evidence in animals has shown that reduced dopaminergic tone may lead to an increase in brainstem excitability, as demonstrated by changes of BRRC^47^. Although several methodological factors may explain the lack of BRRC modification, including the timing of the experiment (i.e. we did not measure BRRC during painful stimulation because it would have been technically challenging) and stimulation intensity, we can conclude that pain-related SBR changes were unlikely due to brainstem excitability modification, but rather to the direct effects of increased central dopaminergic tone modifications of SBR generators.

One hypothesis is that the relationship between central dopaminergic activity and SBR modulation is due to the activation of antinociceptive mechanisms. Dopamine has been shown to play a critical role in antinociceptive processes^18,19^. Both animal and human studies have indicated that dopamine neurons are activated by acute nociceptive stimuli^48–50^ and that dopaminergic response is altered during chronic pain^51^. Positron emission tomography was used to examine the binding of [(11)C]-raclopride (D2/D3 ligand) in the brain during injection of painful hypertonic saline and nonpainful normal saline^51^. While control subjects released dopamine in the basal ganglia during painful stimulation, patients with fibromyalgia experienced hypertonic saline as more painful in comparison to healthy subjects and did not release it^51^. Dopaminergic mimetics showed analgesic effects in tonic pain conditions^52,53^. In addition, the coadministration of either D1-like or D2-like dopaminergic antagonists was able to block µ-opioid receptor-induced antinociception^29^. The hypothesis that SBR modulation due to laser stimuli is due to the activation of antinociceptive mechanisms is also supported by the observation of an inverse correlation between individual SBR changes and subjective pain perception, as assessed by the NRS. As the frequency of spontaneous blinking increased, the perception of pain decreased. In this regard, abnormal pain perception has been observed both in animal models^24,54,55^ and human pathological conditions characterized by a reduction in central dopaminergic tone^16,17,56,57^, such as depression and PD. Moreover, recent pharmacological studies strongly support the role of dopamine in antinociception. Dopamine agonists, like pramipexole, have been shown to induce antiallodynic and antihyperalgesic effects in rats with nigrostriatal lesions^55^. The selective activation of D2 receptors led to decreased nociception, increasing the pain threshold in a transitory or lasting manner according to the experimental conditions. This effect depends on supraspinal mechanisms involving GABA-A and opioid neurotransmission^29^. Conversely, blocking dopamine receptors within the nucleus accumbens inhibited antinociception^30^.

Insight into the relationship between changes of central dopaminergic activity and SBR is provided by another study finding. In this regard, we found that SBR progressively increased during the cold pressor test. The activation of the descending pain modulatory system during the cold pressor test was supported by the increase in the PPT. We admit that SBR modulation does not directly support the role of the descending pain modulatory system, since this effect may be solely due to the tonic nociceptive stimulus. However, considering the temporal profile of SBR modulation, which lasted beyond the end of the stimulus, it is reasonable to hypothesize a possible role of the descending pain modulatory system. Neuroimaging studies demonstrated that the tonic stimulus elicited by the cold pressor test strongly and reproducibly activates the periaqueductal gray and other pain-related brain areas, including the anterior cingulate cortex, thalamus, precentral gyrus, medial frontal gyrus, right inferior frontal gyrus, and left inferior temporal gyrus^17,45^. Our findings pave the way for neurophysiological and functional neuroimaging studies aimed at better understanding the role of dopamine in pain modulation. Central levels of dopaminergic activity may also influence blink reflex^9,58–61^ through brainstem structures that mediate facial reflexes, such as the superior colliculus and nucleus raphe magnus^47,62^.

This study has some limitations. The sample of participants is relatively limited, although the objective techniques we used to quantify blinking movements provided accurate and reproducible measurements^63,64^. In addition, we tested BRRC using only two ISIs. While the assessment of more ISIs could have offered a more accurate evaluation of brainstem excitability, a high number of electrical stimuli delivered at the supraorbital nerve could have induced blink rate habituation^2^. Finally, although SBR is considered a reliable indicator of central dopaminergic tone^3–12^, this parameter can be influenced by numerous factors (e.g. non-dopaminergic system activity, other nonspecific factors including the variable activation of afferent fibers originating from the cornea, the circadian conditions and the state of vigilance)^65–67^. Concerning the latter, although we did not perform an evaluation of the state of vigilance through standardized scales, we minimized this possible confounding factor by performing the experiments in a limited period of time, always visually and verbally checking the state of vigilance of the participants. Moreover, we conducted the experimental procedures at the same time of the day in all subjects.

In conclusion, our results support the role of SBR as a noninvasive biomarker of central dopaminergic tone^3–12^. Most importantly, our study represents the first evidence regarding the use of this noninvasive neurophysiological approach to objectively quantify in vivo the functionality of antinociceptive systems. The results of this study need to be confirmed in further experiments using complementary methodological approaches, including neuroimaging, to investigate nociceptive and antinociceptive systems in humans. Our results offer interesting insights for further investigations concerning pathophysiological pain mechanisms in pathological conditions characterized by dopaminergic system dysfunction, such as depressive states, PD, or schizophrenia, as well as in conditions mainly characterized by pain control system abnormalities, e.g. fibromyalgia^68–70^. Investigating the molecular alterations in dopaminergic systems during different pain conditions may aid in understanding their contribution to the development and maintenance of chronic pain. If confirmed, our approach could be utilized to objectively quantify the antinociceptive effects of analgesic drugs, which could lead to the development of new therapeutic strategies.

## Data Availability

The datasets generated during and/or analysed during the current study are available from the corresponding author on reasonable request.
